# Development of a novel remote‐controlled and self‐contained audiovisual‐aided interactive system for immobilizing claustrophobic patients

**DOI:** 10.1120/jacmp.v16i3.5359

**Published:** 2015-05-08

**Authors:** Harang Ju, Siyong Kim, Paul Read, Daniel Trifiletti, Andrew Harrell, Bruce Libby, Taeho Kim

**Affiliations:** ^1^ Department of Radiation Oncology University of Virginia Health System Charlottesville VA; ^2^ Department of Radiation Oncology Virginia Commonwealth University Richmond VA USA

**Keywords:** audiovisual‐aided interactive, iPad, remote control, self‐contained system, claustrophobic

## Abstract

In radiotherapy, only a few immobilization systems, such as open‐face mask and head mold with a bite plate, are available for claustrophobic patients with a certain degree of discomfort. The purpose of this study was to develop a remote‐controlled and self‐contained audiovisual (AV)‐aided interactive system with the iPad mini with Retina display for intrafractional motion management in brain/H&N (head and neck) radiotherapy for claustrophobic patients. The self‐contained, AV‐aided interactive system utilized two tablet computers: one for AV‐aided interactive guidance for the subject and the other for remote control by an operator. The tablet for audiovisual guidance traced the motion of a colored marker using the built‐in front‐facing camera, and the remote control tablet at the control room used infrastructure Wi‐Fi networks for real‐time communication with the other tablet. In the evaluation, a programmed QUASAR motion phantom was used to test the temporal and positional accuracy and resolution. Position data were also obtained from ten healthy volunteers with and without guidance to evaluate the reduction of intrafractional head motion in simulations of a claustrophobic brain or H&N case. In the phantom study, the temporal and positional resolution was 24 Hz and 0.2 mm. In the volunteer study, the average superior–inferior and right–left displacement was reduced from 1.9 mm to 0.3 mm and from 2.2 mm to 0.2 mm with AV‐aided interactive guidance, respectively. The superior–inferior and right–left positional drift was reduced from 0.5 mm/min to 0.1 mm/min and from 0.4 mm/min to 0.04 mm/min with audiovisual‐aided interactive guidance. This study demonstrated a reduction in intrafractional head motion using a remote‐controlled and self‐contained AV‐aided interactive system of iPad minis with Retina display, easily obtainable and cost‐effective tablet computers. This approach can potentially streamline clinical flow for claustrophobic patients without a head mask and also allows patients to practice self‐motion management before radiation treatment delivery.

PACS number: 87.55.Gh

## I. INTRODUCTION

Patient motion is one of the most prevalent concerns in medical imaging and radiation treatment delivery.^1^, ^2^ If not properly managed during radiotherapy, it can result in insufficient dose delivery to the target and/or unnecessary irradiation of surrounding healthy tissues.^(2)^ A number of diverse motion management techniques for brain/H&N (head and neck) immobilization have been proposed such as the simple face mask, bite block, and more aggressive head frame,^3^, ^4^, ^5^ but most of the systems are intolerable for claustrophobic patients. For example, only a few immobilization systems such as open‐face mask and head mold with a bite plate are available for the claustrophobic patient,^4^, ^6^ but they are mainly mask‐based and make the patients passive recipients of care with a certain degree of discomfort.

Recently, there is evidence that patient interactive (or biofeedback) mechanism can enhance their motion management performance without a patient‐specific immobilization head frame. For example, the concept of patient interactive immobilization for brain/H&N was first introduced by Helmig and Kim,^(7)^ and Kim *et al*.^(8)^ demonstrated feasibility of the concept using a simple laser‐based device for interactive patient H&N immobilization. Similar interactive approaches, under the name of audiovisual (AV) biofeedback, have been widely studied for the management of tumor movement caused by respiratory motion.^9^, ^10^, ^11^ For instance, several research groups demonstrated that AV biofeedback could significantly reduce intrafractional respiratory irregularities during medical imaging or radiation treatment.^9^, ^10^, ^11^ In addition, an interesting concept of quasi‐breath‐hold (QBH) was also possible by virtue of AV biofeedback for breath‐hold motion management.^12^, ^13^

Though these studies have demonstrated improvements in intrafractional motion management including immobilization, an interactive motion management system for claustrophobic patients with H&N cancer is not commercially available. In addition, most systems employ tools that are relatively complicated and costly compared to a head mask, which causes certain extent of limitation in their accessibility by patients and flexibility in clinical use. For example, several commercial systems, such as ExacTrac infrared camera system (BrainLAB, Feldkirchen, Germany), AlignRT (Vision RT, Columbia, IL), and Catalyst (C‐RAD, Uppsala, Sweden), can be utilized for claustrophobic H&N patients. However, their accessibility by patients is limited, and biofeedback is not yet available.

The purpose of this study was to develop and evaluate a novel remote‐controlled and self‐contained audiovisual (AV)‐aided interactive system for immobilizing claustrophobic patients without a head mask, using easily obtainable and cost‐effective tablet computers. Two iPad minis with Retina display were chosen, and mobile applications were developed to provide a more accessible and flexible platform for an AV‐aided interactive system for minimizing intrafractional motion. For feasibility test purposes, the performance of the system was investigated in terms of its resolution, accuracy, and effectiveness on intrafractional head motion management of human subjects simulating a claustrophobic brain or H&N case.

## II. MATERIALS AND METHODS

### A. Self‐contained, AV‐aided interactive system

**Figure 1:**
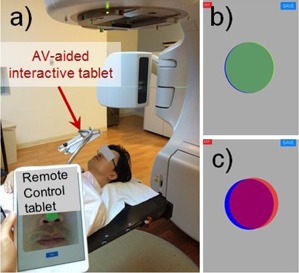
The self‐contained, AV‐aided interactive system of iPad minis with Retina display is shown with the session setup: (a) the AV‐aided interactive tablet and the remote control tablet are shown at the treatment room; (b) a blue disk was shown at the center of the display as a reference for the guiding disk; (c) the color of the circle gradually transitioned from green to red as the motion delta increases from zero to a predetermined warning distance (2 mm for this study).

The system consists of two tablet computers (iPad minis with Retina display, Model A1489; Apple Inc., Cupertino, CA). One unit (iPad mini #1) provides AV‐aided interactive guidance to the subject inside the treatment room, and the other unit (iPad mini #2) is used for remotely controlling the AV‐aided interactive device (iPad mini #1). The system setup is illustrated by Fig. 1. The iPad mini #1 is placed on the device holder below and at around 20 cm anterior–inferiorly away from the subject's head (from the subject's perspective).

For interactive guidance: 1) a marker (colored and nonreflective) is attached on the skin of the patient; 2) the unit #1 continuously captures the image of the marker (in 1280 by 720 resolution using the built‐in front‐facing FaceTime HD camera); 3) image analysis is carried out by the application to determine the position of the marker (consequently that of the patient); and 4) both the current and reference marker positions are displayed so that the patient can react to the feedback in real time.

The marker position is determined by filtering the background based on the marker's color;^(14)^ a green or blue marker is preferred to maximize the signal‐to‐noise ratio. Both image processing and analysis are done with OpenCV for iOS (version 2.4.9; supported by Willow Garage (Menlo Park, Ca) and Itseez (Nizhny Novgorod, Russia)). The calculations are done in pixels first and converted to millimeters. For the pixel‐to‐mm conversion, a mm‐to‐pixel ratio is obtained with the marker's maximum length in pixels measured by the camera and a predetermined length in mm. For example, the millimeter‐to‐pixel ratio was 0.1 mm/pixel at 14 cm and 0.2 mm/pixel at 25 cm. The motion data are then relayed to the subject through AV‐aided interactive guidance in real time on the same tablet unit.

For visual interactive guidance, the tablet displays the right–left and superior–inferior components of the real‐time motion delta by translating a translucent disk on the iPad's display (2048 by 1536 resolution), as shown in Fig. 1. A blue disk is shown at the center of the display as a reference for the guiding disk. To ease the cognitive load of the perception of the motion delta's magnitude, the color of the circle gradually changes from green to red as the motion delta increases from ‘zero’ to a predetermined warning distance (2 mm for this study), as illustrated in Figs. 1(b) and (c).

The audio component of interactive guidance produces short beeps that gradually increase in frequency as the motion delta from the reference point becomes larger. When motion delta is smaller than 10% of the designated warning distance, no beeps are generated. When the motion delta reaches to, and becomes greater than, the designated warning distance, the frequency of beeps plateaus.

### B. Evaluation of basic characteristics

**Figure 2:**
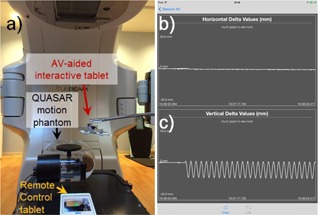
The temporal accuracy and resolution of the system (a) was studied with the QUASAR motion phantom. The AV‐aided interactive tablet measured the right–left (horizontal direction) (b) and superior–inferior (vertical direction) (c) components of real‐time motion and provided the mean displacement in each direction after the session.

The positional accuracy and resolution of the system were investigated through a phantom study using a programmable QUASAR motion phantom (Modus Medical Devices Inc., London, ON, Canada) which is suitable for QA and assessment of motion with submillimeter accuracy, shown in Fig. 2.^15^, ^16^ The marker was moved with three different positions (10 mm, 20 mm, and 30 mm) using the programmed QUASAR motion phantom while the motion was detected and recorded by the tracking device. Tests were made in 15 cm, 30 cm, and 50 cm of the marker plane‐to‐camera distance with parallel (right–left) or perpendicular (superior–inferior) motion.

The temporal accuracy and resolution of the system were studied with the QUASAR motion phantom, which was set to oscillate with a peak‐to‐peak amplitude of 10 mm in a 5 s period. The temporal accuracy was determined by averaging all peak‐to‐peak periods measured by the system.

**Figure 3:**
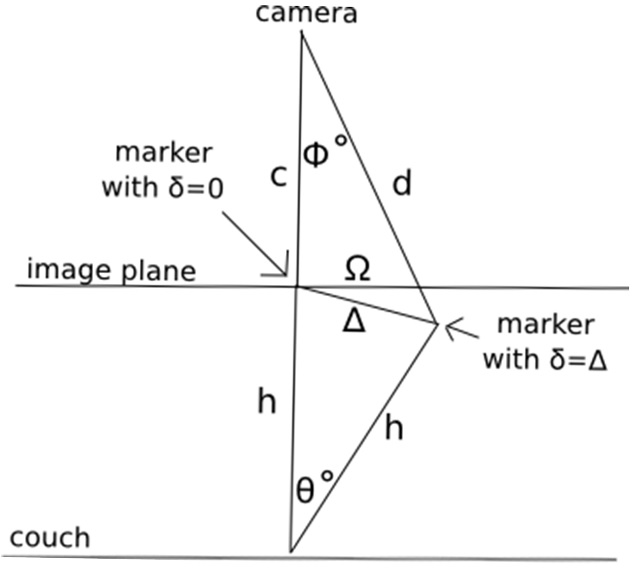
The error resulting from rotational head movements detected by a two‐dimensional camera was simulated in most use cases of the system. A geometric representation of the patient's head rotation with respect to the camera, assuming that the back of the head is anchored by the head rest, where h is the length of the head from the forehead to the back of the head, δ is the symbol of linear displacement, c is the distance from the camera to the marker at δ=0, Δ is the actual linear displacement of the marker caused by the head rotation of θ,Φ is the angle of the camera seeing the displaced marker, d is the distance from the camera to the marker at δ=Δ, and Ω is the calculated displacement by the system.

The error resulting from rotational head movements detected by a two‐dimensional camera was simulated in most use cases of the system. A geometric representation of the patient's head rotation with respect to the camera, assuming that the back of the head is anchored by the head rest, is shown in Fig. 3, where h is the length of the head from the forehead to the back of the head, δ is the symbol of linear displacement, c is the distance from the camera to the marker at δ=0,Δ is the actual linear displacement of the marker caused by the head rotation of θ,Φ is the angle of the camera seeing the displaced marker, *d* is the distance from the camera to the marker at δ=Δ, and Ω is the calculated displacement by the system. It is noted that c, the distance from the camera to the marker at δ=0, is assumed to be 20 cm and h, the length of the head, is assumed to be 20 cm.

### C. Feasibility test with human subjects simulating a claustrophobic brain or H&N case

A feasibility study of intrafractional head motion management for a claustrophobic patient with H&N cancer was performed to investigate the effectiveness of the system. To be conservative, the simulations were carried out under the assumption that patients were severely claustrophobic, meaning that no immobilization device, other than a headrest, was provided.

Ten healthy volunteers (age: 30.2±11.2, age range: 20∼52) participated in the study and their voluntary head motion reductions with AV‐aided interactive guidance were evaluated. Each study consisted of two sessions, one with and the other without guidance to compare the intrafractional head motion. For comparison purposes, the noninteractive session was performed before the AV‐aided interactive session. In the sessions without guidance, the system collected motion data while AV‐aided interactive guidance was not given to the subject. Each session lasted for 5 min in the TrueBeam (Varian Medical Systems, Palo Alto, CA) treatment room or in the CT simulator (Phillips Brilliance Big Bore, Phillips Healthcare, Andover, MA) room. The AV‐aided interactive tablet (unit #1) was placed above the subject's head (using a flexible tablet holder) for AV‐aided interactive guidance, and the tracking marker (green colored) was placed on the nose of the subject, as shown in Fig. 1. The other tablet (unit #2) was utilized for real‐time remote controlling and monitoring (both within and outside the study room) during the session.

The mean displacement was calculated by averaging the absolute values of the displacements (with right–left and superior–inferior data), and the positional drift was calculated by employing a linear fit on the displacements to determine the mm per min drift. Quantitative statistical comparison of mean right–left and superior–inferior displacements, 95% confidence interval, and positional drift between the two sessions was performed using the paired Student's *t*‐test and evaluated in a spreadsheet program (Excel 2010, Microsoft, Redmond, WA).

## III. RESULTS

### A. Basic characteristics

The mean variation in positional accuracy was 0.3 mm, 0.8 mm, and 0.8 mm, and the mean positional resolution was 0.2 mm, 0.3 mm, and 0.5 mm with 15 cm, 30 cm, and 50 cm of the marker plane‐to‐camera distance, respectively. The mean variation in temporal accuracy was less than 0.6 ms, and the mean temporal resolution was 24 Hz (42 ms per data point, as shown in Fig. 2).

Three angular displacements of 0.28°, 1.43°, and 2.86° rotation in the simulation, as illustrated in Fig. 3, caused 1 mm, 5 mm, and 10 mm translational motion, respectively. On the image plane, the distances detected by the camera were 0.99 mm, 4.99 mm, and 9.98 mm, respectively, resulting in not larger than 0.02 mm error within the range of angular displacement tested.

### B. Feasibility test with human subjects simulating a claustrophobic brain or H&N case

**Figure 4:**
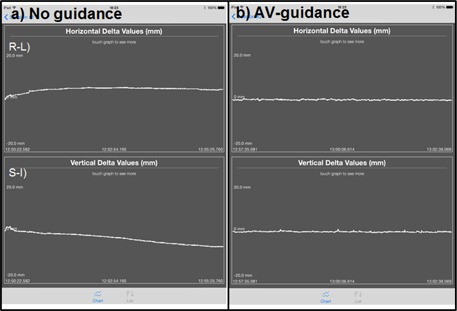
Intrafractional voluntary head motions of the volunteer 10 (representative of the 90th percentile range of reduced the mean displacement due to AV‐aided interactive guidance) are shown (a) without AV‐aided interactive guidance and (b) with AV‐aided interactive guidance. The mean displacement decreased superior–inferiorly (SI direction) by an average of 94% (from 3.1 mm to 0.2 mm) and right–left direction (RL direction) by an average of 95% (from 4.4 mm to 0.2 mm) with AV‐aided interactive guidance.

Table 1 shows average displacement and baseline drift and results of paired Student *t*‐test comparing the immobilizing methods. In the table, the results are that intrafractional voluntary head motion, on average, was kept within 0.3±0.2 mmfor the superior–inferior direction with guidance (Fig. 4), showing promising performance, while that without guidance was 1.9±1.9 mm (p−value=0.015). In addition, intrafractional voluntary head motion in the right–left direction was reduced from 2.2±1.3 mmwithout guidance to 0.2±0.1 mmwith guidance (Fig. 4) (p−value<0.001). Compared to the case of no‐guidance, the mean displacement was decreased by an average of 88% in the superior–inferior direction and by an average of 91% in the right–left direction.

**Table 1:** Means displacement ± standard deviation (STD), baseline drift of the right‐left and superior‐inferior motion, and paired Student t‐test p‐values with / without AV‐aided interactive guidance.

*Motion Direction*		Mean Displacement±STD (mm)	*95% CI*	*p‐value*	*Baseline Drift (mm/min)*	*p‐value*
Right–Left	No guidance	2.2±1.3	0∼4.8		0.4±0.3	
	AV‐guidance	0.2±0.1 (−91%)	0∼0.4	<0.001	0.4±0.02 (−91%)	0.003
Superior– Inferior	No guidance	1.9±1.9	0∼5.7		0.5±0.5	
	AV‐guidance	0.3±0.2 (−88%)	0∼0.7	0.015	0.1±0.2 (−80%)	0.06

Noticeable positional drift was also observed in the sessions without AV‐aided interactive guidance, while the head position of the volunteers remained almost unchanged with guidance presented in Table 1. The average drift was decreased with AV‐aided interactive guidance from 0.5±0.5 mm/min to 0.1±0.2 mm/min (80% reduction, p–value=0.06) in the superior–inferior direction and from 0.4±0.3 mm/min to 0.04±0.02 mm/min (91% reduction, p−value=0.003) in the right–left direction.

## IV. DISCUSSION

In this study, a remote‐controlled and self‐contained audiovisual‐aided interactive system of iPad minis with Retina display was developed for claustrophobic patients with H&N cancer, and its resolution and accuracy, as well as its efficacy when applied to human subjects, were evaluated.

This system provides benefits as a self‐contained system of tablet computers. In the study, intrafractional voluntary head motion was reduced from 1.9 mm to 0.3 mm for the superior–inferior direction with guidance, suggesting the reduction of the CTV‐PTV margins if the motion is properly managed. Another benefit is that it can be easily accessible by patients, and they could potentially practice AV‐aided interactive guidance using their own personal device(s) at the waiting room or even at home. Note that the AV‐aided interactive tablet can be used with complete functionality without the remote control tablet. In addition, any iOS device, including iPhone and iPod, can substitute both the AV‐aided interactive and remote control components. Due to the widespread commercial availability and use of smart devices, such as iOS devices, the volunteers were already familiar with the devices and had no trouble using them. The system is simple to use and set up and, thus, it can be implemented without difficulty in most clinics, even including places where resources are limited. As mentioned earlier, if the system is installed in waiting areas for patients to practice before they begin radiation therapy treatments or CT simulation, it could improve not only delivery accuracy but also workflow efficiency.

The system utilizes the tablet's built‐in, front‐facing camera to detect motion and consequently is constrained by the inherent resolution and limitations of the camera. The system showed an error range of one pixel, and the corresponding error in millimeters depended on the distance from the camera to the marker. For example, the millimeter‐to‐pixel ratio was 0.2 mm/pixel at 15 cm and 0.3 mm/pixel at 30 cm. At greater distances, the positional resolution of the system would decrease. In practice, however, it is not likely to have such long distance. As long as the camera is within 50 cm, under 1 mm resolution can be obtained.

The main limitations of the system are that the 2D motion tracking method of the current system does not include motion in the third translational dimension (anterior–posterior direction) and rotational errors. However, the headrest or additional head supports secure the head position in the anterior–posterior direction. In addition, the error resulting from rotational head movements detected by a two‐dimensional camera is not significant, if the rotational motion is not huge. For example, as can be observed from Fig. 3, head displacement of around 10 mm caused by a 3° rotation would be detected within a relative error of about 0.2% by the system (at 20 cm away from the marker).

In this study, the system tracked a single marker on the subject's open face and detected two translational degrees of freedom. Specifically, the marker was placed on the nose assuming that the nose provides a prominent and relatively fixed area of the face for visual tracking. In a three‐dimensional space, three translational planes and three rotational axes exist; thus, the two‐dimensional motion detection of the system did not reflect true three‐dimensional motion. However, the tablet was close in distance to the subject's head, and the subjects were limited in their movements by the physiology of the human head and neck in supine position. Therefore, we believe, two‐dimensional motion detection would be sufficient for intrafractional head motion reduction in the current use case. Although a test is not included in this study, the system can readily track multiple markers on different areas of the face and display the mean displacement of them to obtain better accuracy, if needed. Furthermore, in principle, multi‐body‐part tracking can be realized using multiple units and markers placed on various body parts. In most head‐and‐neck cases, for instance, not only the head but also other relevant body parts, such as shoulders and neck, are important. Therefore, their positions can be identified and corrected during initial image guidance, and a mask on the neck and shoulder can help the patient remain in place. However, if a claustrophobic patient cannot tolerate a mask on the neck and shoulder, additional monitoring may be necessary; two additional iPads can be arranged for real‐time monitoring, one for the neck and the other for the shoulders.

In the simulation of the study, the iPad was positioned anterior–inferiorly to the patient's face during treatment, to monitor the whole brain motion and to avoid direct irradiation to the iPad. In cases, however, some beam angles with couch kicks in IMRT or VMAT treatments interfere with the current iPad position, and so the iPad can be placed more inferiorly with the aid of visual accessories, such as reflecting glasses.

This system consists of two tablets, one for motion tracing and audiovisual guidance, and the other for remote control. With a router outside the treatment room wired to an access point inside the room, an infrastructure Wi‐Fi can be created and both tablets can communicate with each other between the treatment and control rooms. The AV‐aided interactive tablet streams frames captured from the front‐facing camera to the remote control tablet and can potentially transmit other useful information that can be easily monitored by the remote control tablet. One of this system's key benefits is that only a single small mobile device is required in the treatment room for AV‐aided interactive guidance, and the remote control device can communicate with the AV‐aided interactive tablet anywhere outside of the treatment room.

The iPad close to the beam in the treatment room would be damaged by radiation. To our best knowledge, unfortunately, no systematic study of radiation damage on the iPad has been reported. Considering that planned replacement of iPad can be easily executed due to both the abundant availability and cost‐effectiveness of the device, we believe radiation damage would not be a critical issue. However, further investigations are needed.

To bring the proposed system into the clinic, a couple of potential issues need to be considered. First, the proposed 2D monitoring system needs to be verified with an independent system, such as 2D or 3D radiographic imaging. Although the calculations illustrated in Fig. 3 show that head displacement of around 10 mm with a 3° head rotation would be detected within a relative error of about 0.2%, verification with an independent imaging system can provide practical guidelines on the use of the proposed system. Second, the audiovisual‐aided guidance method used in this study may not be optimal for actual patients, who may have visual or auditory impairments. Therefore, an audiovisual‐aided guidance that depends on the visual and auditory capabilities of the specific patient needs to be determined prior to the procedure.

Compared to the most commercially available motion management systems, such as ExacTrac infrared camera system, AlignRT Real‐time Positioning Management (RPM) system, and Active Breath Hold (ABC) system with limited accessibility by patients, the proposed system is cost‐effective. As a result, its unique and sufficient functionality of AV‐aided interactive guidance in the treatment room, as well as in the waiting room, can be utilized without substantial financial burden.

## V. CONCLUSIONS

This study developed and demonstrated a self‐contained AV‐aided interactive system for claustrophobic patients with brain or H&N cancer using easily obtainable and cost‐effective tablet computers only (iPad mini with Retina display). This approach can potentially streamline clinical flow for claustrophobic patients without a head mask and by allowing patients to practice self‐motion management before radiation treatment delivery. To bring the proposed 2D tracking system into the clinic, it needs to be verified with an independent 2D or 3D imaging system.

## ACKNOWLEDGMENTS

This study was supported by George Amorino Pilot Grant in Radiation Oncology at University of Virginia Health System.

## Supporting information

Supplementary Material

Supplementary Material

Supplementary Material
