# Supplementary figures and images for: Development of a novel remote‐controlled and self‐contained audiovisual‐aided interactive system for immobilizing claustrophobic patients
Source: J Appl Clin Med Phys. 2015 May 8;16(3):216–24. doi: 10.1120/jacmp.v16i3.5359 (PMC5690133; doi:10.1120/jacmp.v16i3.5359)

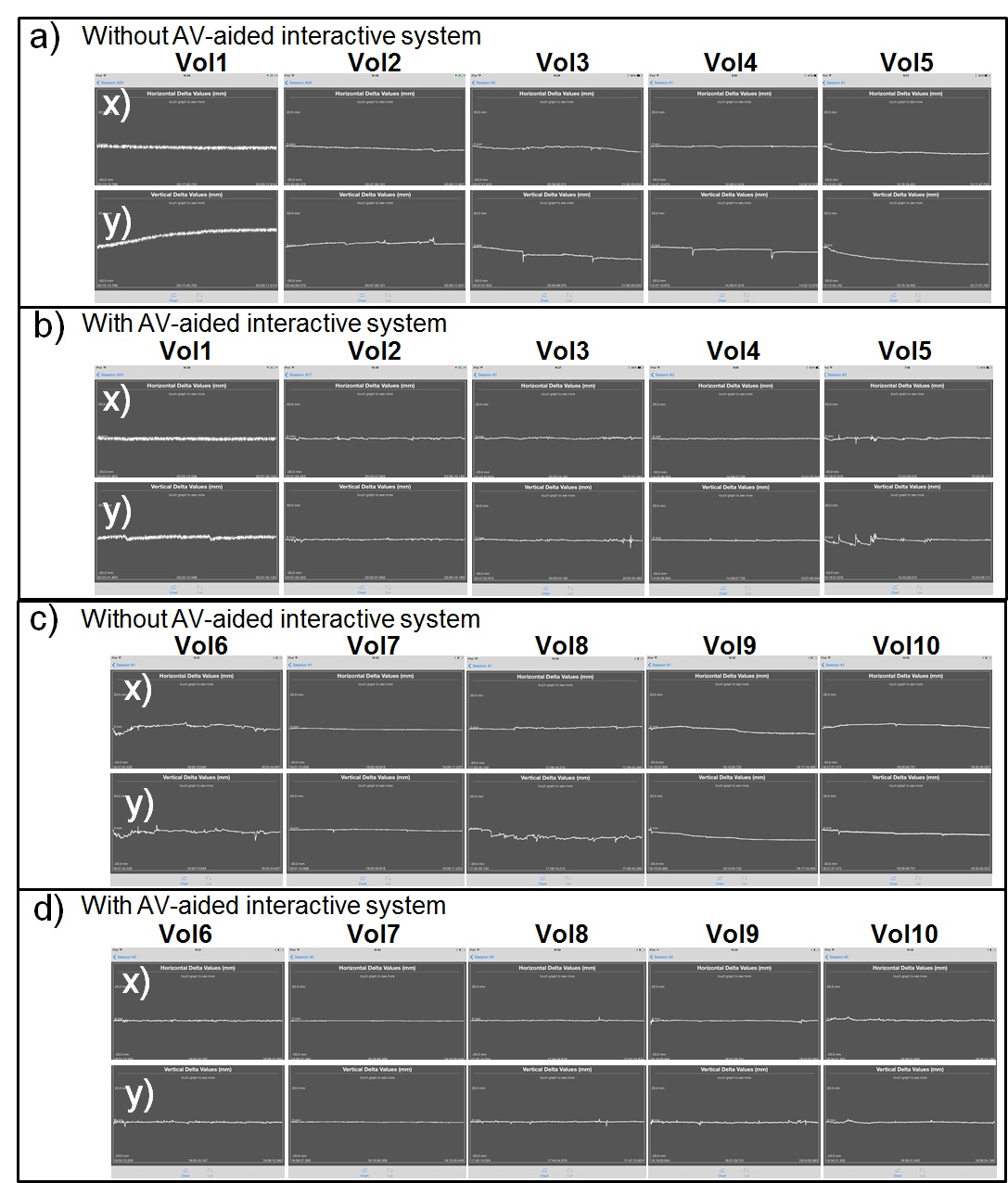

Supplement: Supplementary file 1 — Supplementary Material [file ACM2-16-216-s001.jpg]

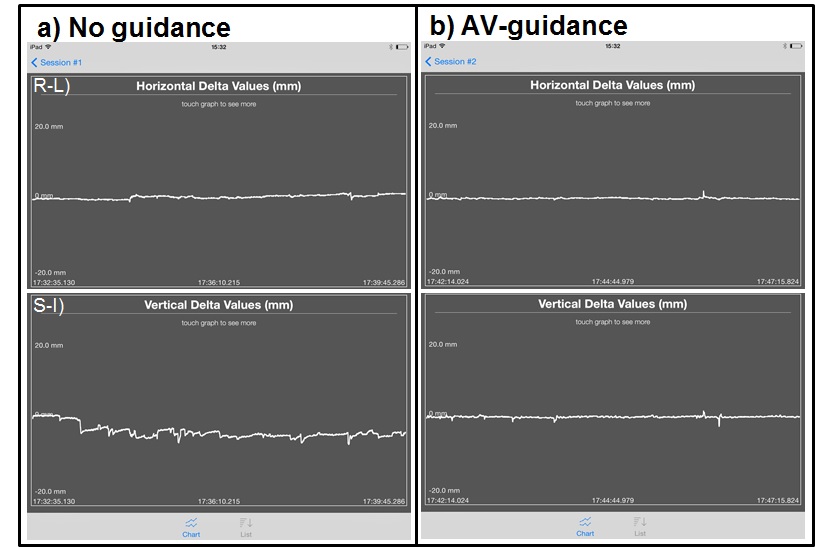

Supplement: Supplementary file 2 — Supplementary Material [file ACM2-16-216-s002.jpg]

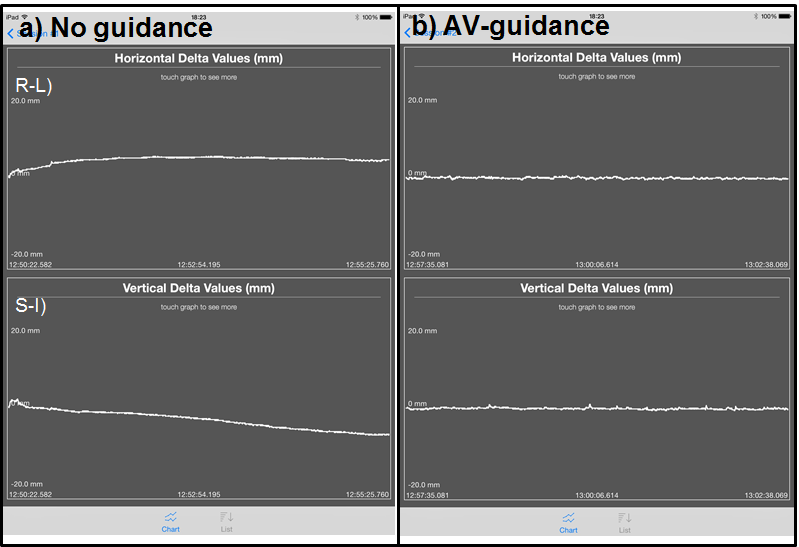

Supplement: Supplementary file 3 — Supplementary Material [file ACM2-16-216-s003.tif]
